# WISDoM: Characterizing Neurological Time Series With the Wishart Distribution

**DOI:** 10.3389/fninf.2020.611762

**Published:** 2021-01-26

**Authors:** Carlo Mengucci, Daniel Remondini, Gastone Castellani, Enrico Giampieri

**Affiliations:** ^1^Department of Physics and Astronomy, University of Bologna, Bologna, Italy; ^2^Department of Agricultural and Food Sciences, University of Bologna, Bologna, Italy; ^3^Department of Experimental, Diagnostic and Specialty Medicine, University of Bologna, Bologna, Italy

**Keywords:** supervised learning, time series, null model, feature selection, classification, Wishart distribution, uneven sampling

## Abstract

WISDoM (Wishart Distributed Matrices) is a framework for the quantification of deviation of symmetric positive-definite matrices associated with experimental samples, such as covariance or correlation matrices, from expected ones governed by the Wishart distribution. WISDoM can be applied to tasks of *supervised learning*, like classification, in particular when such matrices are generated by data of different dimensionality (e.g., time series with same number of variables but different time sampling). We show the application of the method in two different scenarios. The first is the ranking of features associated with electro encephalogram (EEG) data with a time series design, providing a theoretically sound approach for this type of studies. The second is the classification of autistic subjects of the Autism Brain Imaging Data Exchange study using brain connectivity measurements.

## 1. Introduction

High-dimensionality time-structured data are extremely common in fields such as finance, biophysics, and biomedical data. Very often, experimental limitations lead to uneven sampling (i.e., a different number of time points in terms of frequency or duration) (Bazzani et al., 2010) and this poses problems for many types of analysis (e.g., sample classification). As a consequence, clipping or padding techniques are applied, altering the underlying temporal structure. In recent years, studies on such data have seen an increasing popularity in a wide range of fields, from functional magnetic resonance imaging (fMRI) (Van Den Heuvel and Pol, 2010; Azarmi et al., 2019; Yan et al., 2019) to time series exploration for critical transition prediction in clinical scenarios (Cuesta-Frau et al., 2019; Ghalati et al., 2019). The common goal of this type of research is to develop models and algorithms capable of reaching the highest possible classification and prediction performances, for diagnostic and real-time applications, while unveiling underlying information about a system. Reproducibility and generalization issues of commonly applied methods are in part caused by *ad-hoc* preprocessing of data, due to the lack of simple null models, often substituted by reshuffling-based null models. We introduce a method based on the statistical distribution of symmetric positive-definite matrices (i.e., covariance and correlation matrices) extracted from data, using the Wishart distribution as a null model, as a possible way to overcome some of the aforementioned issues. Properties of distribution of random symmetric positive-definite matrices have proven to be useful in fields such as condensed matter, especially in the study of disordered systems (Crisanti et al., 1994; Zhu et al., 2009). The WISDoM method exploits the properties of the Wishart distribution in order to compute limit distributions for the classes of samples in a classification problem, and a log-likelihood based score is defined for the single variables to quantify their relevance in the classification task.

## 2. Methods

### 2.1. The Wishart Distribution

The *Wishart distribution W*_*p*_(*n*, Σ) is a probability distribution of random non-negative-definite *p* × *p* matrices that is used to model random covariance matrices.

The parameter *n* is the number of degrees of freedom (e.g., the number of points in the time series), and Σ is a non-negative-definite symmetric *p* × *p* matrix (with *p* the number of variables, or *features*, of the time series) called the *scale matrix*.

*Definition*. Let *X*_1_…*X*_*n*_ be *N*_*p*_(0, Σ) distributed vectors, forming a data matrix *p* × *n*, *X* = [*X*_1_…*X*_*n*_]. The distribution of a *p* × *p*, $M=XX^{\prime }=\Sigma_{i=1}^{n}X_{i}X_{i}^{\prime }$ *random matrix* is a Wishart distribution (Hardle and Simar, 2003).

We have then by definition:

(1) $$\begin{array}{r} M\sim W_{p}\left(n,\Sigma \right)\sim \Sigma_{i=1}^{n}X_{i}X_{i}^{\prime }\;X_{i}\sim N_{p}\left(0,\Sigma \right) \end{array}$$

so that *M* ~ *W*_*p*_(*n*, Σ) is the distribution of a sum of *n* rank-one matrices defined by independent normal $X_{i}\in R^{p}$ with *E*(*X*) = 0 and *Cov*(*X*) = Σ.

In particular, it holds for the present case:

(2) $$\begin{array}{r} E\left(M\right)=nE\left(X_{i}X_{i}^{\prime }\right)=nCov\left(X_{i}\right)=n\Sigma \end{array}$$

### 2.2. PDF Computation for Invertible Σ

In general, any *X* ~ *N*(μ, Σ) can be represented as

(3) $$\begin{array}{r} X=\mu +AZ,\;Z\sim N\left(0,I_{p}\right) \end{array}$$

so that

(4) $$\begin{array}{r} \Sigma =Cov\left(X\right)=ACov\left(Z\right)A^{\prime }=AA^{\prime } \end{array}$$

The easiest way to find *A* in terms of Σ is the LU-decomposition, which finds a unique lower diagonal matrix *A* with *A*_*ii*_ ⩾ 0 such that *AA*′ = Σ.

Then by Equations (1) and (4), with μ = 0 we have:

(5) $$\begin{array}{r} W_{p}\left(n,\Sigma \right)\sim \overset{n}{\underset{i=1}{\sum }}\left(AZ_{i}\right){\left(AZ_{i}\right)}^{\prime }\sim A\left(\overset{n}{\underset{i=1}{\sum }}Z_{i}Z_{i}^{\prime }\right)A^{\prime }\sim AW_{p}\left(n\right)A^{\prime } \end{array}$$

where *Z*_*i*_ ~ *N*(0, *I*_*p*_) and *W*_*p*_(*n*) = *W*_*p*_(*I*_*p*_, *n*).

Assuming that *n* ≥ *p* and Σ is invertible, the density of the random *p* × *p* matrix *M* in 1 can be written as

(6) $$\begin{array}{r} f\left(M,n,\Sigma \right)=\frac{1}{2^{\frac{np}{2}}\Gamma_{p}\left(\frac{n}{2}\right)|\Sigma {|}^{\frac{n}{2}}}|M{|}^{\frac{n-p-1}{2}}exp\left[-\frac{1}{2}tr\left(\Sigma^{-1}M\right)\right] \end{array}$$

so that *f*(*M, n*, Σ) = 0 unless *M* is *symmetric and positive-definite* (Anderson et al., 2012).

Note that in Equation (6), we define Γ_*p*_(α) as the *generalized gamma function*:

(7) $$\begin{array}{r} \Gamma_{p}\left(\alpha \right)=\pi^{\frac{p\left(p-1\right)}{4}}\overset{p}{\underset{i=1}{\prod }}\Gamma \left(\frac{2\alpha +1-i}{2}\right) \end{array}$$

### 2.3. Estimation of the Wishart Parameters From Empirical Covariance

We justify the use of the Wishart distribution under the assumption of multivariate Gaussian distributed data scenarios. This kind of assumption is generally good for a wide range of problems. With an adequate sample size, distributions with non-divergent second-order moment converge to a Gaussian distribution, due to central limit theorem, and the estimation of the covariance matrix still yields. In addition, preprocessing strategies (such as Box–Cox or logarithmic transform) can be applied to non-Gauss distributed data. As far as non-stationarity in time series data are concerned, which could affect the assumptions required for our approach, these issues can be addressed by applying common global or detrending strategies (such as LOESS). Furthermore, the use of the average covariance matrix (obtained from all the elements of one class) to compute the scale matrix for the class estimated distribution will be proven to be a good approximation of a complete Bayesian model.

This is done by considering that the Wishart Distribution is the conjugate prior of a multivariate Gaussian distribution, such as the gamma distribution for the univariate Gaussian case. By considering a Gaussian model with known mean μ, so that the free parameter is the variance σ^2^, as in Liu and Wasserman (2014), the likelihood function is defined as follows:

(8) $$\begin{array}{r} p\left(X_{1}...X_{n}|\sigma^{2}\right)\propto {\left(\sigma^{2}\right)}^{-\frac{n}{2}}exp\left(-\frac{1}{2\sigma^{2}}n\bar{{\left(X-\mu \right)}^{2}}\right), \end{array}$$

(9) $$\begin{array}{r} \bar{{\left(X-\mu \right)}^{2}}=\frac{1}{n}\overset{n}{\underset{i=1}{\sum }}{\left(X_{i}-\mu \right)}^{2} \end{array}$$

The conjugate prior is an inverse Gamma distribution. Recall that θ has an inverse gamma distribution with parameters (α, β) when $\frac{1}{\theta }\sim Gamma\left(\alpha ,\beta \right)$.

The density then takes the form

(10) $$\begin{array}{r} \pi_{\alpha ,\beta }\left(\theta \right)\propto \theta^{-\left(\alpha +1\right)}e^{-\frac{\beta }{\theta }} \end{array}$$

Using this prior, the posterior distribution of σ^2^ is given by

(11) $$\begin{array}{r} p\left(\sigma^{2}|X_{1}...X_{n}\right)\sim InvGamma\left(\alpha +\frac{n}{2},\beta +\frac{n}{2}\bar{{\left(X-\mu \right)}^{2}}\right) \end{array}$$

In the multidimensional setting, the inverse Wishart takes the place of the inverse gamma. It has already been stated that the Wishart distribution is a distribution over *symmetric positive semi-definite d* × *d* matrices *W*. A more compact form of the density is given by

(12) $$\begin{array}{r} \pi_{\nu_{0},S_{0}}\left(W\right)\propto |W{|}^{\frac{\left(\nu_{0}-d-1\right)}{2}}exp\left(-\frac{1}{2}trace\left(S_{0}^{-1}W\right)\right), \end{array}$$

(13) $$\begin{array}{r} |W|=det\left(W\right) \end{array}$$

where the parameters are the degrees of freedom ν_0_ and the positive-definite *scale matrix S*_0_.

If $W^{-1}\sim Wishart\left(\nu_{0},S_{0}\right)$, we can then state that *W* has an *Inverse Wishart Distribution*, whose density has the form

(14) $$\begin{array}{r} \pi_{\nu_{0},S_{0}}\left(W\right)\propto |W{|}^{-\frac{\left(\nu_{0}+d+1\right)}{2}}exp\left(-\frac{1}{2}trace\left(S_{0}W^{-1}\right)\right), \end{array}$$

Let *X*_1_…*X*_*n*_ be *N*(0, Σ) distributed observed data. Then an inverse Wishart prior multiplying the likelihood *p*(*X*_1_…*X*_*n*_∣Σ) yields

(15) $$\begin{array}{r} p\left(X_{1}...X_{n}|\Sigma \right)\pi_{\nu_{0},S_{0}}\left(\Sigma \right)\propto \end{array}$$

(16) $$\begin{array}{r} |\Sigma {|}^{-\frac{n}{2}}exp(-\frac{n}{2}tr(\bar{S}\Sigma^{-1})|\Sigma {|}^{-\frac{\left(\nu_{0}+d+1\right)}{2}}exp\left(-\frac{1}{2}tr\left(S_{0}\Sigma^{-1}\right)\right) \end{array}$$

(17) $$\begin{array}{r} =|\Sigma {|}^{-\frac{\left(\nu_{0}+d+n+1\right)}{2}}exp\left(-\frac{1}{2}tr\left(\left(n\bar{S}+S_{0}\right)\Sigma^{-1}\right)\right) \end{array}$$

where $\bar{S}$ is the *empirical covariance*:

(18) $$\begin{array}{r} \bar{S}=\frac{1}{n}\overset{n}{\underset{i=1}{\sum }}X_{i}X_{i}^{T} \end{array}$$

Thus, an a posteriori distribution with the form

(19) $$\begin{array}{r} p\left(\Sigma |X_{1}...X_{n}\right)\sim InvWishart\left(\nu_{0}+n,n\bar{S}+S_{0}\right) \end{array}$$

is obtained.

Similarly, it can be stated that for the inverse covariance (*precision*) matrix Σ^−1^ the conjugate prior is a Wishart distribution.

### 2.4. Classwise Estimated Distribution

The core idea of the WISDoM method is to represent each element undergoing classification as a covariance matrix of its features. Nominally, each element can be characterized by the covariance matrix extracted by the repeated observations of the vector of its features, for example derived by a time series. The aim is to use the *free parameters* of the Wishart distribution (the scale matrix *S*_0_ and the number *n* of the degrees of freedom, as shown in 6) to compute an estimation of the distribution for a certain class of elements, and then assign a single element to a given class by computing a log-likelihood between the element being analyzed and each class. Furthermore, a score can be assigned to each feature by estimating the variation in terms of log-likelihood, due to its removal from the feature set. If the removal of a feature causes significant increase (or decrease) in the log-likelihood, it can be stated that such feature is highly representative of the system analyzed. Thus, the WISDoM approach allows not only to assign a given element to a class, but also to identify the features with the highest relevance in the classification process.

Covariance matrices are a good choice for a distance metrics in a classification task, both for the way they represent a system and for the property that the average of a set of covariance matrices is a covariance matrix itself. If each element of a given class *C* is represented by a covariance matrix Σ of its features, being *N*_*C*_ the number of the elements belonging to class *C*, this property allows us to estimate a distribution for the class by choosing

(20) $$\begin{array}{r} S_{0}={\hat{\Sigma }}_{C}=\frac{1}{N_{C}}\overset{N_{C}}{\underset{i=1}{\sum }}\Sigma_{i} \end{array}$$

The other necessary parameter for the estimation is the *number of degrees of freedom n*. Assume that an *X*_*i*_ = (*x*_1_, …, *x*_*p*_) vector of *p* features is associated with each element *i* of a given class, while having *n* observations for this vector. The covariance matrix Σ_*i*_ computed over the *n* observations will represent the interactions between the features of element *i*. The number of degrees of freedom *n* of the Wishart distribution is then given by the number of times *X*_*i*_ is observed.

Let us give an example tied to *functional MR brain imaging*. An image of patient *i*'s brain is acquired; as usual these images are divided in a certain number *p* of zones (voxel, pixel, etc.), each zone being sampled *n* times over a given time interval in order to observe a certain type of brain activity and functionality. In this example, the features contained in vector *X*_*i*_ = (*x*_1_, .., *x*_*p*_) associated with patient *i* are indeed the zones chosen to divide the brain image, each zone having been sampled *n* times during an acquisition interval. The *p* × *p* correlation matrix Σ_*i*_ is then representative of the functional correlation between the *p* brain areas. Repeating this procedure for the *N*_*C*_ patients of a known class *C* (i.e., a diagnostic group) and computing the ${\hat{\Sigma }}_{C}$ scale matrix for the class will allow us to estimate a Wishart distribution for that class and draw samples from it.

### 2.5. Log-Likelihood Ratio Score

After defining how to represent classes distribution, WISDoM allows to compute the log-likelihood of each element to belong to one of the classes. Moreover, WISDoM allows to compute the variation of log-likelihood ratio scores due to the removal of features, singularly or in groups, thus estimating how much the classification performance changes. Uninformative (or less informative) features can thus be pruned, allowing for a dimensionality reduction of the initial feature set. The whole process can be seen as a feature transformation, mapping the covariance matrix Σ_*i*_ of subject *i* to a score vector formed by the change in log-likelihood for each feature.

#### 2.5.1. Complete Matrix Score

The WISDoM Classifier relies upon computing the log-likelihood of a matrix Σ_*i*_ with respect to the Wishart distribution estimated for a class *C* using ${\hat{\Sigma }}_{C}$ as the scale matrix. If a problem concerning two given classes *C*_*A*_ and *C*_*B*_ is taken into account, the score assigned to each Σ_*i*_ can be defined as follows:

(21) $$\begin{array}{r} score_{i}=logP_{W}\left(\Sigma_{i}|n,{\hat{\Sigma }}_{A}\right)-logP_{W}\left(\Sigma_{i}|n,{\hat{\Sigma }}_{B}\right) \end{array}$$

where ${\hat{\Sigma }}_{A,B}$ are the scale matrices computed for the classes *A, B*, respectively, and $logP_{W}\left(\Sigma_{i}|n,{\hat{\Sigma }}_{A,B}\right)$ is the logarithm of the probability of Σ_*i*_ belonging to the Wishart distribution estimated for one of the two classes *A, B*.

#### 2.5.2. Single Feature Score

WISDoM allows to obtain information about the features used for classification by reducing the matrix *A* to its *principal submatrices* (see Supplemental Materials). An important property for the principal submatrices of a symmetric positive definite matrix is that *any* (*n* − *k*) × (*n* − *k*) *partition is also symmetric and positive definite*.

By removing one feature from the dataset, calculating the WISDoM scores, and iterating this process over all the features (i.e., analyzing all the (*p* − 1) × (*p* − 1) principal submatrices of Σ_*i*_ and ${\hat{\Sigma }}_{C}$) the method can assign a score to each feature, representing its relevance in the decision for Σ_*i*_ to be assigned to one class or another. Let Σ_*j*_ be a principal submatrix of order (*p* − 1) of the matrix Σ computed on the observation of *X*_*i*_ = (*x*_1_, …, *x*_*p*_) for subject *i*, *obtained by the deletion of the j*^*th*^ *row and the j*^*th*^ *column*. Similarly, let ${\hat{\Sigma }}_{Cj}$ be a principal submatrix of order (*p* − 1) of the matrix ${\hat{\Sigma }}_{C}$ computed for the class *C*. The score assigned to each feature of *X*_*i*_ = (*x*_1_, …, *x*_*p*_) is then given by Equation (23).

(22) $$\begin{array}{r} Score_{j}\left(C\right)=\Delta logP_{Wj}\left(C\right)= \end{array}$$

(23) $$\begin{array}{r} logP_{W}\left(\Sigma ,n|{\hat{\Sigma }}_{C},n\right)-logP_{W}\left(\Sigma_{j},n|{\hat{\Sigma }}_{Cj},n\right) \end{array}$$

In a 2-class example, we obtain a score vector as follows:

(24) $$\begin{array}{r} Ratio_{j}=\Delta logP_{Wj}\left(C_{1}\right)-\Delta logP_{Wj}\left(C_{2}\right) \end{array}$$

A generalization to (*p* − *n*) dimensionality reduction can be found in the Supplementary Materials.

## 3. Results

### 3.1. Eye State Detection via EEG

**Figure 1:**
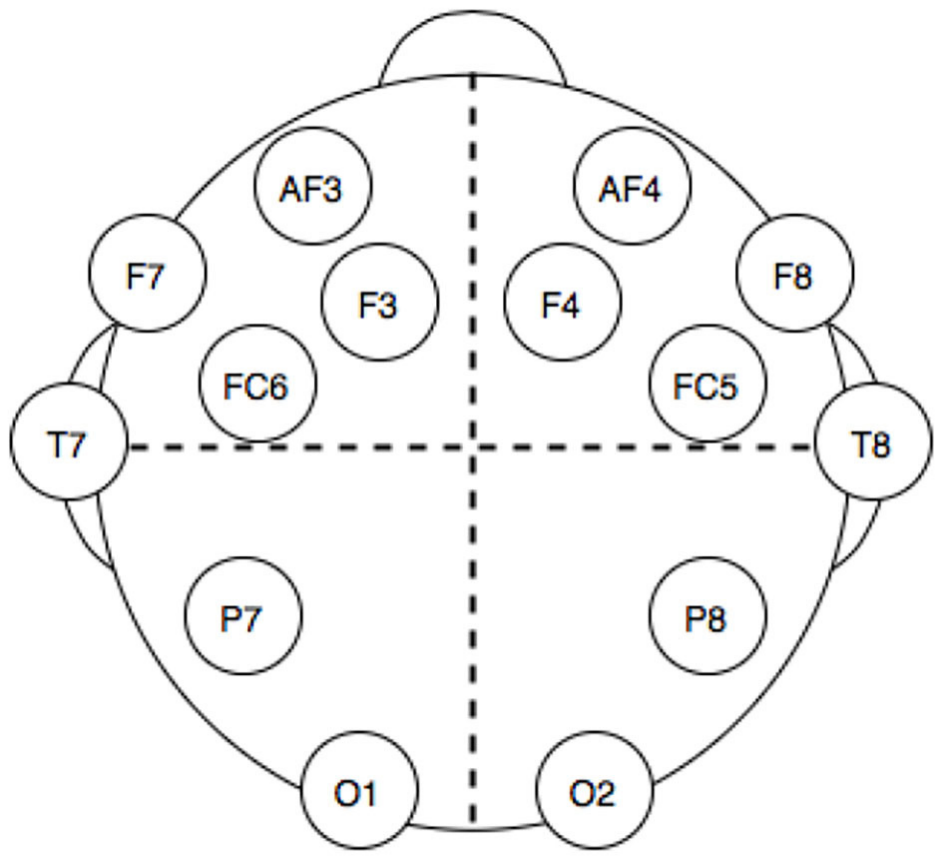
Electrodes position in the headset used for electro encephalogram (EEG) dataset acquisition.

The dataset used was downloaded from the UCI Machine Learning Repository (http://archive.ics.uci.edu/ml/). This dataset has been chosen for many reasons: it is openly accessible and contains records from 14 electrodes with standard headset placement (Figure 1), thus making the features of our problem directly linked to brain topology and a published classification performance benchmark on the dataset exists (Rajesh et al., 2015). The data consisted of a series of 14,980 time points, sampled for each one of the 14 electrodes and labeled with a 1 or a 0 to mark whether the eyes of the subject are open or closed at that time point. The time series has been split into batches of different length according to eye state changes. In this way, a correlation matrix can be extracted for each batch (the “elements” for this classification problem), while the length of each batch is used for computing the degrees of freedom of each class Wishart distribution during training. A total of 140 batches with various lengths, 70 with eye state 1, and 70 with eye state 0, were obtained.

The representative matrix for each class is computed as the average (weighted on the length of each batch) of matrices of the elements belonging to eye state 0 or eye state 1, excluding the element to be predicted in a *Leave One Out* fashion in order to avoid overfitting. By doing this, we verify that the method is independent from the sampling window chosen when applied to time series data, with the only constraint that the length of such window cannot be less than the number of the features of the system.

After undergoing feature score computation, a stochastic grid search on a set of classifiers has been performed in order to obtain the best prediction performance with the transformed features. All the classification tasks are validated through a *10-fold cross-validation*. Versions and references for all Python packages used can be found in Supplementary Materials and various other studies (Jones et al., 2001; Travis, 2006; Hunter, 2007; Perez and Granger, 2007; McKinney, 2010; Kluyver et al., 2016; Meurer et al., 2017; Waskom et al., 2017).

**Figure 2:**
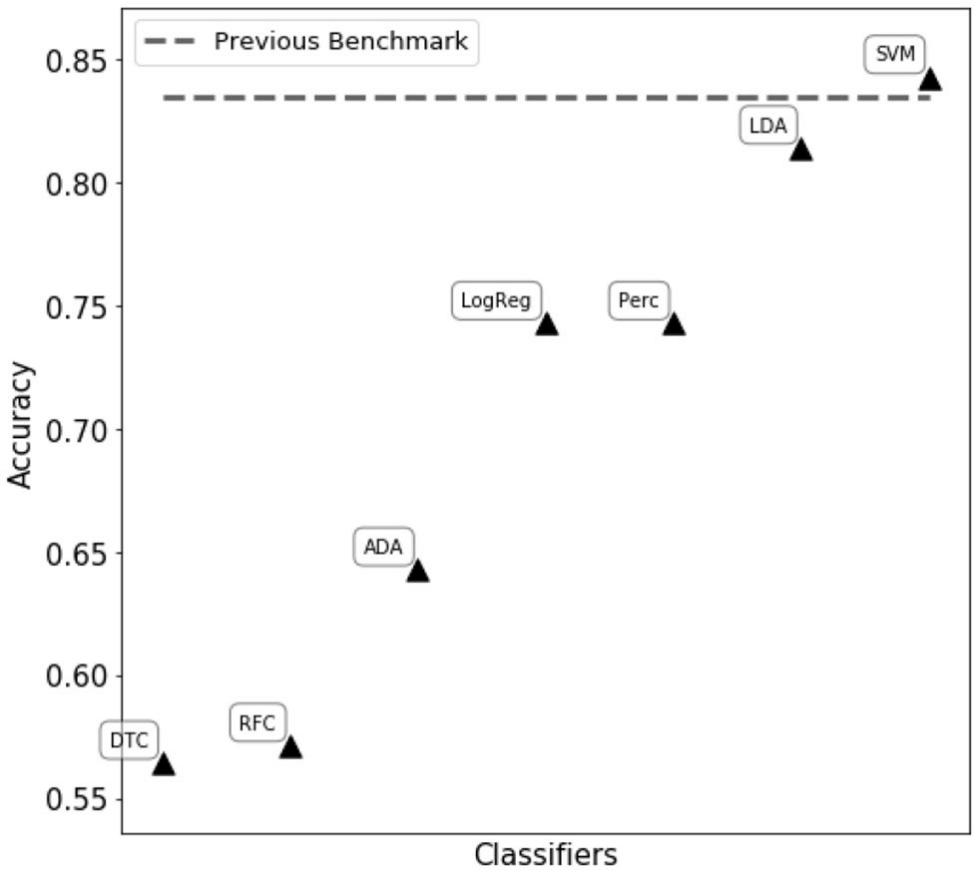
Performance comparison of different classifiers on the WISDoM electro encephalogram (EEG)-transformed features. The classifiers are reported as follows: RFC, Random Forest Classifier (Breiman, 2001); DTC, Decision Tree Classifier (Breiman et al., 1984; Barros Rodrigo et al., 2012); ADA, ADA Boosting Tree Classifier (Freund and Schapire, 1997; Zhu et al., 2009); LDA, Linear Discriminant Analysis Classification (McLachlan, 2004); LogReg, Logistic Regression Classifier (Hsiang-Fu et al., 2011); Perc, Perceptron Classifier (Freund and Schapire, 1999); SVM, C-Support Vector Machine (Platt, 1999; Chang and Lin, 2013). All classifiers are SciKitLearn implementations.

We first tried to assess eye state using complete matrix score, as given in Equation (21). Classifiers reported in Figure 2 were trained and tuned, with the aim of obtaining the best performance possible. However, in this scenario the resulting classification performances were poor, reaching an accuracy of ~ 60% in the best cases. We then proceeded to compute single feature scoring, as given in Equation (23), obtaining a feature transformation. As shown in Figure 2, different classifiers belonging to two main categories (decision trees and linear classifiers) have been trained on the transformed features. The best performance has been achieved with a C-support Vector Machine (*Python 3.6 SciKitLearn implementation)* resulting in a 0.85% ROC AUC score and an accuracy score of 84.3%, comparable with the benchmark of 83.5% accuracy set by Rajesh et al. (2015).

**Figure 3:**
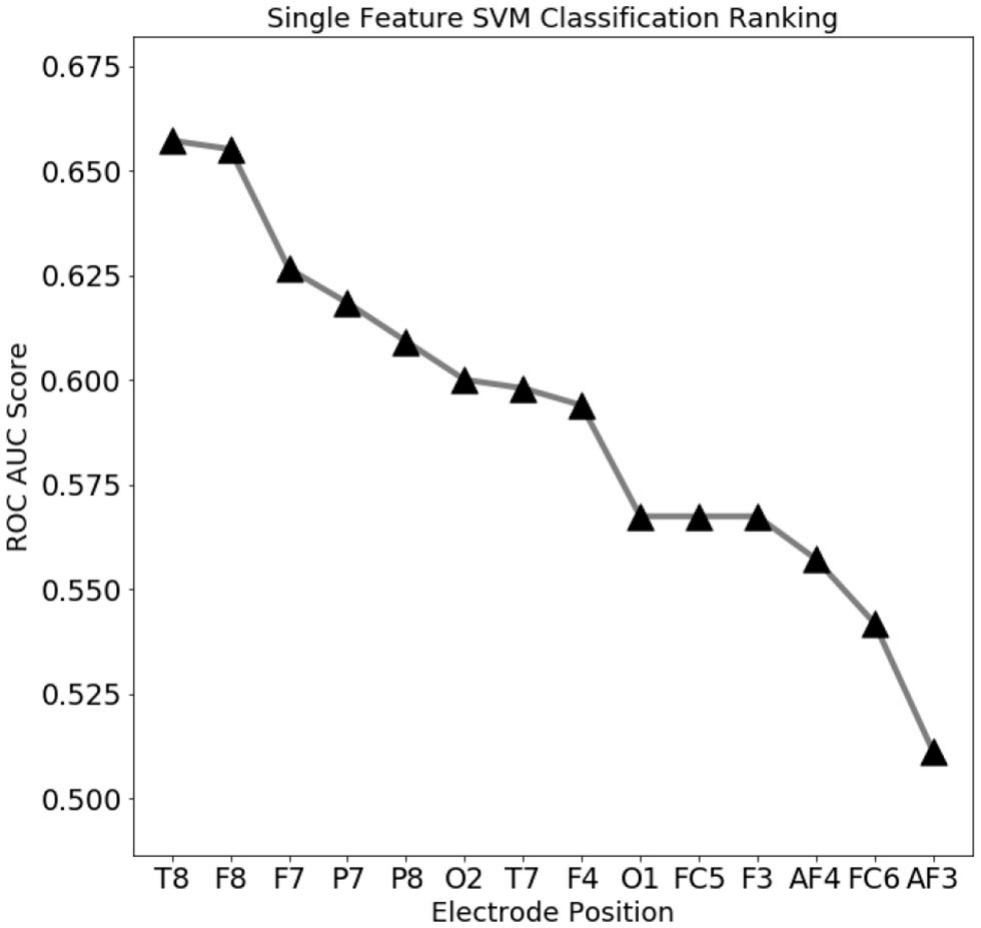
Electro encephalogram (EEG) feature ranking based classification performance (ROC AUC score). Temporal (T8) and outer frontal (F8, F7) electrodes seems to convey the most important signals for eye state prediction.

**Figure 4:**
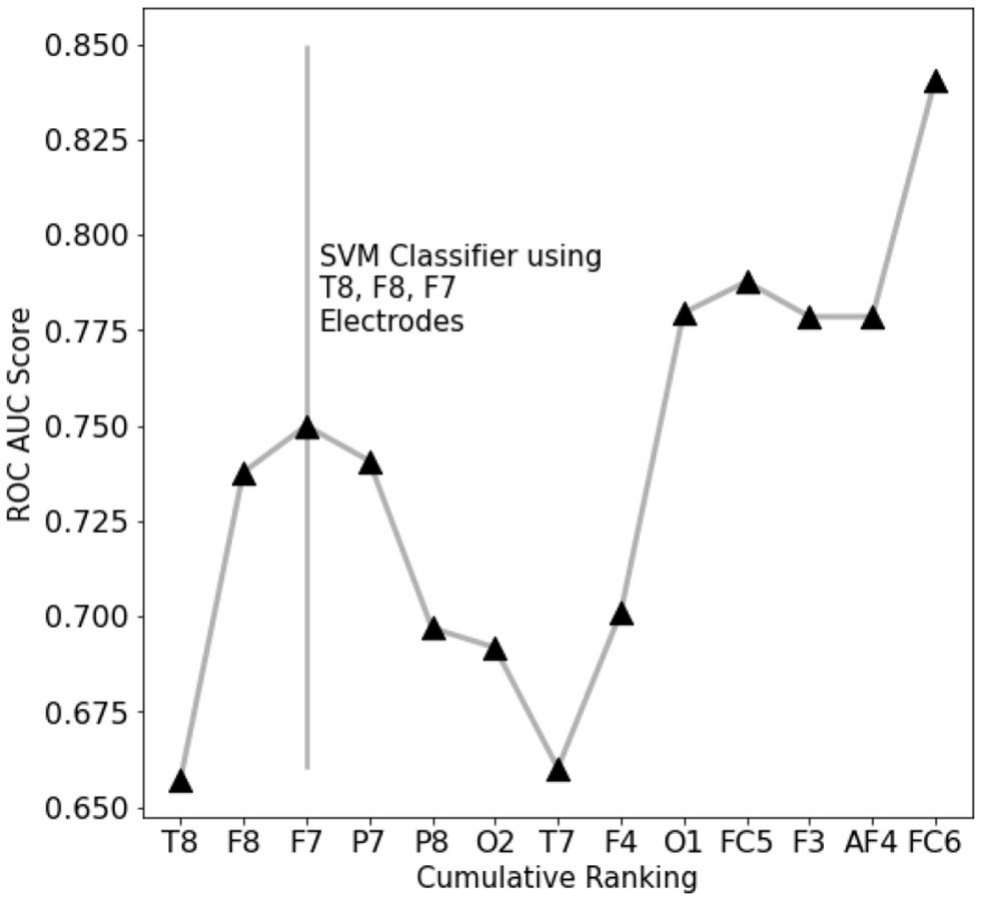
EEG features performance landscape at an increasing number of ranked features used for classification. Labels on the X-axis point out which feature is being added to the previous ones.

To assess which features contain the largest amount of useful information for prediction, a set of single-feature C-SVM classification has been performed (Figure 3): a performance of 75% accuracy is obtained by using only the top three ranking electrodes (Figure 4). Training the classifier with the top three electrodes yields a local maxima in the landscape performance, highlighting the importance of the information recorded by these three electrodes about the state of the whole system.

**Figure 5:**
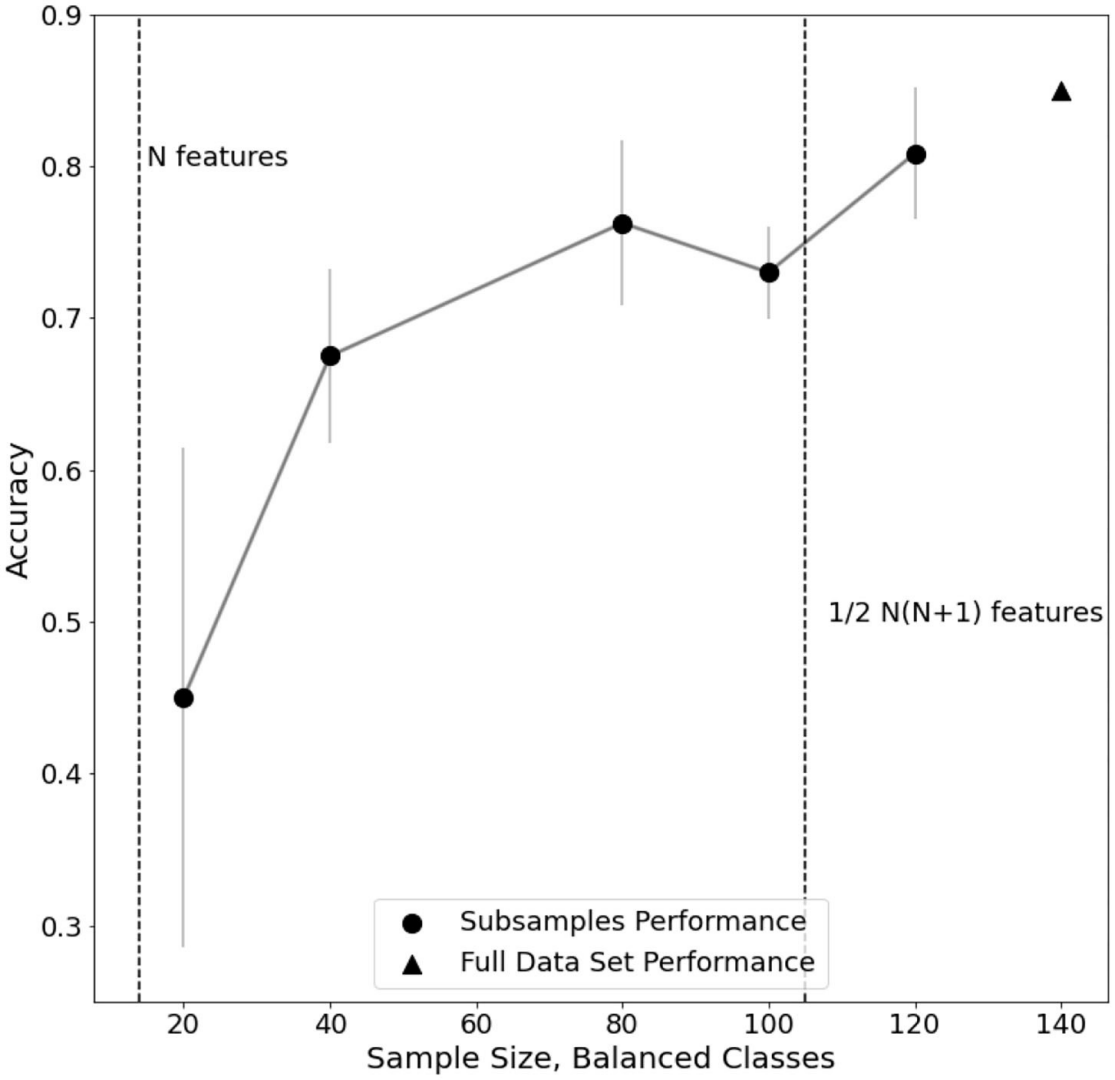
Evaluation of performance trend on subsamples of electro encephalogram (EEG) dataset. For each subsample, the standard error of a fivefold cross-validated C-SVM classification is reported.

Furthermore, an analysis of classification performance as a function of sample size is reported (Figure 5). Equally sized randomized subsamples of each class are extracted for feature transformation and SVM classification training. Classification performance and stability rapidly drop when approaching subsamples of size comparable with the number of features in the EEG dataset, highlighting the need of adequate statistics for the method to be reliable.

### 3.2. Autism Classification via fMRI

The Autism Brain Imaging Data Exchange (ABIDE) (Di Martino et al., 2014) is a consortium effort, aggregating fMRI datasets from individuals with autism spectrum disorders (ASD) and age-matched typical controls (TC). Data from 17 acquisition sites were merged using different preprocessing tools and pipelines (Craddock et al., 2013). Complete information about the dataset is found at *http://preprocessed-connectomes-project.org/abide/*. For our classification task, we focused on male subjects of the “Autism” diagnostic group (AUT): we analyzed a total of 369 TC and 220 AUT subjects, with 200 time points each (number of degrees of freedom of the Wishart distribution). The chosen preprocessing pipeline for the extraction of the average time series of the regions of interest (ROIs) is the Connectome Computation System (CCS), with a global signal correction and the application of a band-pass filter (0.01–0.1 Hz). The 116 ROIs (features), of which covariance matrices are extracted, are labeled according to the Automated Anatomical Labeling of the Brain (AAL2) (Tzourio-Mazoyer et al., 2002).

The representative matrix for each class is computed as the average of matrices of the elements belonging to class AUT or TC, excluding the sets of element to be predicted. For this task, a shuffled 10-fold splitting of the dataset for feature score computation has been used to avoid overfitting. After undergoing feature score computation, the parameters of a C-Support Vector Machine Classifier have been fine tuned in order to obtain the best prediction performance with the transformed features. All the classification tasks are validated through a *stratified 10-fold cross-validation* in order to minimize the effects of class imbalance in train and test sets. Versions and references for all Python packages used can be found in Supplementary Materials and in Jones et al. (2001), McKinney (2010), Hunter (2007), Travis (2006), Meurer et al. (2017), Kluyver et al. (2016), Perez and Granger (2007), and Waskom et al. (2017).

**Figure 6:**
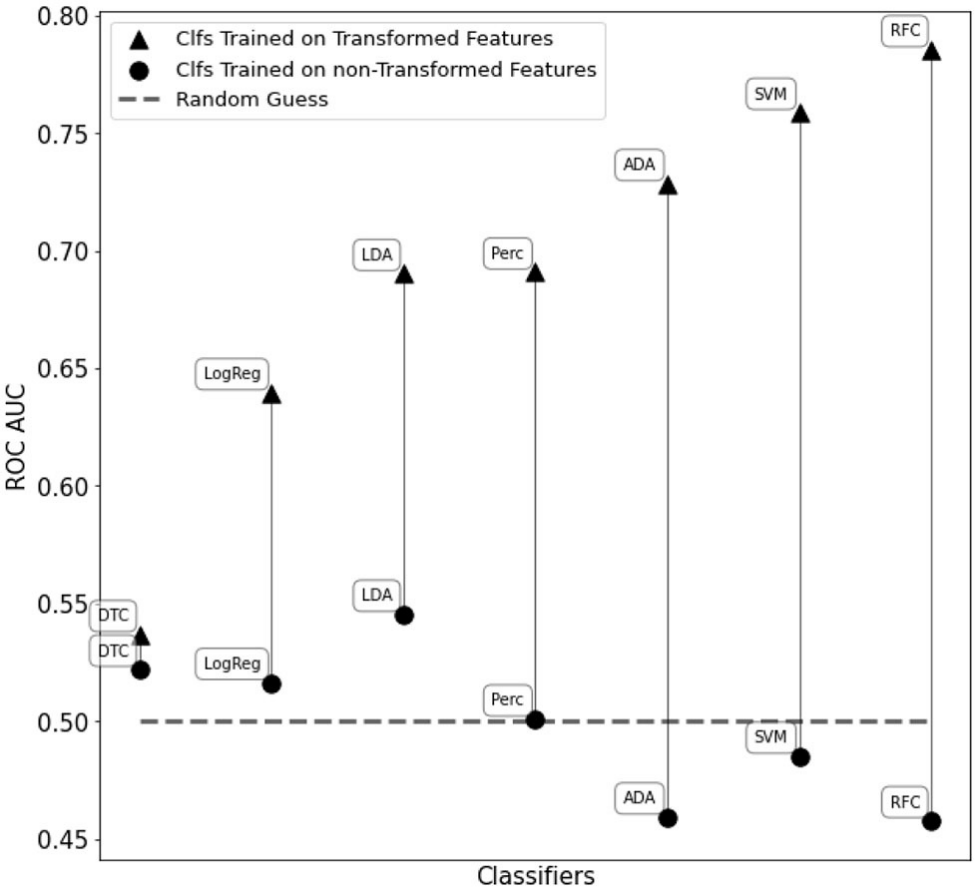
Performance comparison of different classifiers on the WISDoM transformed features and non-transformed feature in the ABIDE dataset. The classifiers are reported as follows: RFC, Random Forest Classifier (Breiman, 2001); DTC, Decision Tree Classifier (Breiman et al., 1984; Barros Rodrigo et al., 2012); ADA, ADA Boosting Tree Classifier (Freund and Schapire, 1997; Zhu et al., 2009); LDA, Linear Discriminant Analysis Classification (McLachlan, 2004); LogReg, Logistic Regression Classifier (Hsiang-Fu et al., 2011); Perc, Perceptron Classifier (Freund and Schapire, 1999); SVM, C-Support Vector Machine (Platt, 1999; Chang and Lin, 2013). All classifiers are SciKitLearn implementations.

The C-SVM classifier trained on the transformed features resulted in an accuracy score of 72.1% and an ROC AUC score of 0.76. Furthermore, we obtained a ROC AUC score of 0.79 and an accuracy of 73.5% with a fine-tuned Random Forest Classifier. We also compared the classifiers in Figure 2 training them with WISDoM transformed features and non-transformed features (nominally, the elements of the lower triangle of each covariance matrix). Results in Figure 6 show an overall improvement of classification performances when using transformed features. As a comparison, the state of art of classification on the entire ABIDE dataset is set at 70% accuracy obtained with a deep learning architecture built by Heinsfeld et al. (2017). This result on the whole spectrum of autism required the use of various stacked denoising autoencoders and hidden layers, resulting in a large time-consuming training routine (~33 h), while WISDoM obtained satisfying classification performance in much smaller time (~18 min, including feature transformation that is the most time-consuming step of the pipeline).

## 4. Discussion

The WISDoM framework is introduced: a method for modeling symmetric positive definite matrices, such as covariance and correlation matrices, used in a wide array of problems. It can provide a null model for classification purposes in which each sample is represented as a covariance/correlation matrix, even if the number of observations (e.g., the length of the time series) is different from sample to sample. This property makes the WISDoM method suitable for problems with non-homogeneous data size, for example time series with uneven lengths, missing points or irregularly sampled data. Moreover, we show that a feature transformation based on WISDoM scores can be used for dimensionality reduction, providing a ranking for the most important variables in the dataset. While showing good generalization capabilities with time-series data and non-homogeneous sampling-related issues, the method is not suitable when the number of features exceeds the sampling (*p* > *n*). This is a theoretical limit tied to the invertibility of the scale matrix required to compute the Wishart probability density function. At present, WISDoM cannot thus be applied to problem involving the so-called “wide data,” such as gene expression tables, unless considering corrections such as matrix regularization methods and hierarchical methods such as power priors (Ibrahim et al., 2015).

The method has been tested on the EEG eye state prediction dataset of the open *UCI Machine Learning Repository*, slightly improving the previous classification benchmark with little to no preprocessing, and giving useful insights on the minimum number and location of electrodes needed to record sufficient information for the task. Moreover, the method has been applied to the classification of a subset of the ABIDE dataset using brain functional connectivity data. We obtained satisfying classification scores, comparable with the state of art classification results on the dataset, with very simple classifiers and without the use of additional time-consuming processing routines. Furthermore, the Bayesian-like framework of scores-computation through log-likelihood could allow for a sort of inline learning by continuously updating the estimation of each class Wishart distribution. This property makes the WISDoM method also suitable for real-time learning during data acquisition.

## Data Availability Statement

Publicly available datasets were analyzed in this study. This data can be found here: (1) UCI Machine Learning Repository http://archive.ics.uci.edu/ml/, (2) Autism Brain Imaging Data Exchange http://preprocessed-connectomes-project.org/abide/.

## Author Contributions

EG, CM, GC, and DR designed the research. CM and EG analyzed the data and implemented the method. CM, DR, GC, and EG wrote the paper. All authors contributed to the article and approved the submitted version.

## Conflict of Interest

The authors declare that the research was conducted in the absence of any commercial or financial relationships that could be construed as a potential conflict of interest.
