# Supplementary material for: WISDoM: Characterizing Neurological Time Series With the Wishart Distribution
Source: Front Neuroinform. 2021 Jan 26;14:611762. doi: 10.3389/fninf.2020.611762 (PMC7875084; doi:10.3389/fninf.2020.611762)
Supplement: Supplementary file 1 [file Data_Sheet_1.PDF]

Supplementary Material

1 APPENDIX

1.1 Visualizing the Wishart Distribution

The Wishart distribution is a generalization to multiple dimensions of the *chi-squared distribution*, or in the case of non-integer degrees of freedom, of the *gamma distribution*.

We show in fig.S1 that for a 1-dimensional and equal to 1 Σ scale matrix, the Wishart distribution $W_1(n, 1)$ is equivalent to the $\chi^2(n)$ distribution.

Save for this simple case, being the Wishart a distribution over matrices, it is a generally hard task to visualize it as a density function. Samples can be however drawn from it and the eigenvectors and eigenvalues of the resulting sampled matrix can be exploited to define an ellipse.

An example of this technique is shown in fig.S2. A set of five sampled matrices is drawn for each plot. The upper plots show sampling for Wishart distributions with $n_{degfreedom} = 80$, while the lower plots show sampling for Wishart distributions with $n_{degfreedom} = 10$ in order to show the effects of varying both the scale matrix and the number of degrees of freedom. Note that for $\Sigma = I_2$ (left plot in fig.S2) the sample would look *on average* like circles. The scale matrix for the right-most plot is $\Sigma = \begin{bmatrix} 4 & 3 \\ 3 & 4 \end{bmatrix}$

1.2 Principal Submatrices

Def. *Let A be an $n \times n$ matrix. A $k \times k$ submatrix of A formed by deleting $n - k$ rows of A, and the same $n - k$ columns of A, is called principal submatrix of A. The determinant of a principal submatrix of A is called a principal minor of A.*

Note that the definition does not specify which $n - k$ rows and columns to delete, only that their indices must be the same.

Let us introduce a 3×3 example.

For a general matrix $A_{3\times3}$

$$A = \begin{bmatrix} a_{11} & a_{12} & a_{13} \\ a_{21} & a_{22} & a_{23} \\ a_{31} & a_{32} & a_{33} \end{bmatrix} \tag{S1}$$

there are three *first order principal minors*:

- $| a_{11} |$ formed by deleting the last two rows and columns
- $| a_{22} |$ formed by deleting the first and third rows and columns
- $| a_{33} |$ formed by deleting the first two rows and columns

There are three *second order principal minors*:

- $| \begin{bmatrix} a_{11} & a_{12} \\ a_{21} & a_{22} \end{bmatrix} |$ formed by deleting column 3 and row 3

- $| \begin{bmatrix} a_{11} & a_{13} \\ a_{31} & a_{33} \end{bmatrix} |$ formed by deleting column 2 and row 2
- $| \begin{bmatrix} a_{22} & a_{23} \\ a_{32} & a_{33} \end{bmatrix} |$ formed by deleting column 1 and row 1

There's one *third order principal minor*, namely $\mid A \mid$.

For the sake of completion, we also recall the following definition.

Def. *Let A by an $n \times n$ matrix. The kth order principal sub-matrix of A obtained by deleting the* ***last*** *$n - k$ rows and columns of A is called the kth order* ***leading principal submatrix*** *of A, and its determinant is called the kth* ***order leading principal minor*** *of A.*

1.3 Generalizing to $(p - n)$ Order Transformations

Transforming all the $(p-1) \times (p-1)$ principal submatrices of Σ_i by eq.(22), yields a vector of score of length p for each element i. Anyway, for any $n < p$, a number of principal submatrices of Σ_i can be obtained. These kind of submatrices can be used to gain information about the weight of n simoultaneously deleted features on the system structure and classification. Let us introduce an example for $(p-2)$ order submatrices. Let Σ_{jk} be a principal submatrix of order $(p-2)$, of the matrix Σ_i computed on the observation of $X_i = (x_1, ..., x_p)$ for subject i, *obtained by the deletion of the jth row and the jth column and the kth row and the kth column* , with $1 \leq j, k \leq p$. Let $\hat{\Sigma}_{Cjk}$ be a principal submatrix of order $(p-2)$, of the matrix $\hat{\Sigma}_{Cjk}$ computed for the class C *obtained by the deletion of the jth row and the jth column and the kth row and the kth column* , with $1 \leq j, k \leq p$.

Then, eq.(22) becomes:

$$Score_{jk}(C) = \Delta logP_{Wjk}(C) = \tag{S2}$$

$$logP_W(\Sigma, n \mid \hat{\Sigma}_C, n) - logP_W(\Sigma_{jk}, n \mid \hat{\Sigma}_{Cjk}, n) \tag{S3}$$

in this case, a score is assigned to each coupling of the features j, k, and transformation (22) will yield not a vector, but a $p \times p$ matrix with diagonal elements equals to the scores obtained by (22), being the iteration with $j = k$ the coupling of the jth feature with itself. Non-diagonal elements represent the score of the coupling of feature j with feature k. A notable characteristic of $(p-2)$ order transformations for correaltion matrices, is that the informative content after such an order of transformation is comparable to that of the original correlation matrix. It can thus be stated that *the $(p-2)$ order transformed correlation matrix seems to be equivalent to the original correlation matrix under an affine transformation.*

1.4 Figures

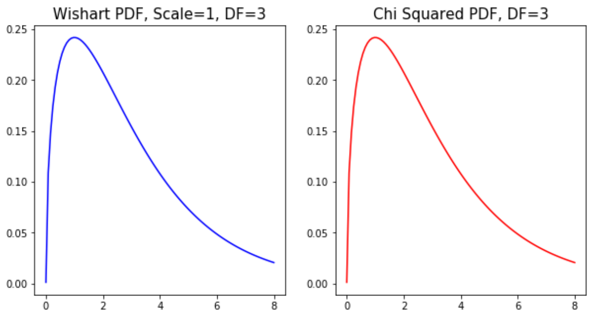

Figure S1. *Monodimensional Wishart Distribution and $\chi^2(n)$ distribution comparison*

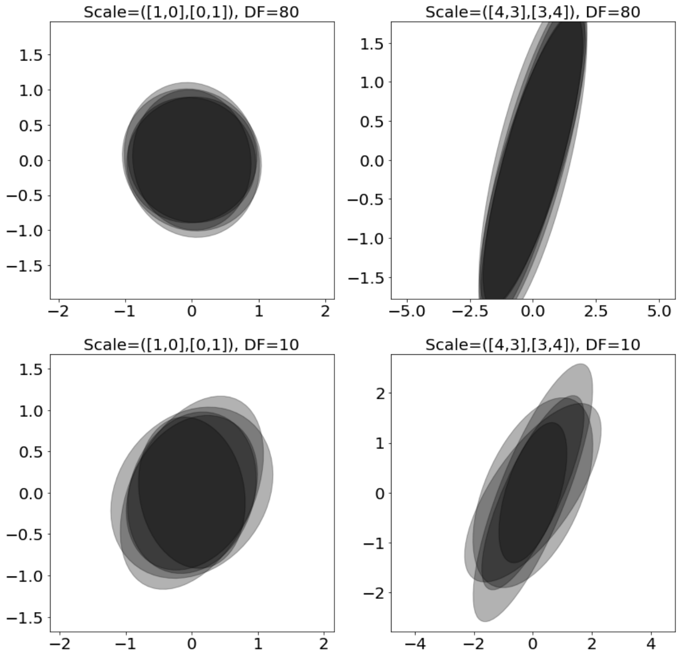

Figure S2. *Plot of eigenvalue and eigenvectors defined ellipses, drawn from different Wishart distributions.*

2 SOFTWARE VERSIONS

- Python Version 3.6.2
- SciPy Version 0.19.14
- Pandas Version 0.25.3
- MatPlotLib Version 3.0.3
- NumPy Version 1.13.3
- SimPy Version 3.0.11
- Seaborn Version 0.9.0
